# Sexual function after voluntary medical male circumcision for human immunodeficiency virus prevention: Results from a programmatic delivery setting in Botswana

**DOI:** 10.4102/sajhivmed.v21i1.1042

**Published:** 2020-04-20

**Authors:** Jillian C. Pintye, Kathleen E. Wirth, Conrad Ntsuape, Nora J. Kleinman, Lisa Spees, Bazghina-werq Semo, Shreshth Mawandia, Jenny Ledikwe

**Affiliations:** 1 Department of Global Health, University of Washington, Seattle, United States; 2 Botswana International Training and Education Center for Health (I-TECH), Gaborone, Botswana; 3 Department of Biostatistics, Harvard T.H. Chan School of Public Health, Boston, Massachusetts, United States; 4 Department of HIV/AIDS Prevention and Care, Botswana Ministry of Health and Wellness, Gaborone, Botswana; 5 NJK Consulting, Seattle, Washington, United States; 6 Amgen Asia Holdings Ltd, Hong Kong, Japan; 7 Department of Health Policy and Management, University of North Carolina, Chapel Hill, United States,; 8 Lineberger Comprehensive Cancer Center, University of North Carolina, Chapel Hill, United States; 9 FHI 360, Washington, Washington, United States

**Keywords:** Botswana, voluntary medical male circumcision, human immunodeficiency virus (HIV) prevention, men, implementation science, program delivery

## Abstract

**Background:**

Uptake of voluntary medical male circumcision (VMMC) remains modest in Botswana in spite of the government’s commitment and service provision availability. Data on sexual function post-VMMC in programmatic settings could help guide messaging tailored to Botswana.

**Objectives:**

At 3-month post-VMMC, we evaluated changes in sexual function and satisfaction with the VMMC procedure amongst a cohort of HIV-negative, sexually active men aged 18–49 years who underwent VMMC in a public-sector clinic in Botswana.

**Methods:**

We assessed whether each of the following domains of sexual function had improved, stayed the same or worsened since VMMC: sexual desire, ability to use condoms, ease of vaginal penetration, ease of ejaculation, ability to achieve and maintain an erection and hygiene or cleanliness.

**Results:**

Data on sexual function were available for 378 men at 3-month post-VMMC. Median age was 27 years – 54% had a higher than secondary education, 72% were employed and 27% were married. Nearly all (96%) the men reported improvement in at least one domain of sexual function, while 19% reported improvement in all six domains. One-fourth (91/378, 24%) of the men reported that at least one domain of sexual function worsened post-VMMC. The most frequently reported domain that worsened was sexual desire (11%); in all other domains, < 10% of the men reported worsening. Men who reported any worsening sexual function were 2.3-fold as likely to be less than ‘very satisfied’ with the VMMC procedure (risk ratio 2.36, 95% confidence interval [CI] 1.66–3.34, *p* < 0.001).

**Conclusion:**

Emphasising improved sexual function experienced after VMMC in demand-creation efforts could potentially increase VMMC uptake in Botswana.

## Introduction

Continued promotion of voluntary medical male circumcision (VMMC) programmes in countries with high human immunodeficiency virus (HIV) burden and low male circumcision rates is needed to decrease population-level HIV incidences.^1,2,3^ More than 18.6 million men have been circumcised through VMMC programmes in 14 priority African countries to date, averting an estimated 230,000 new HIV infections by 2017.^4,5,6,7,8^ Achieving the global target of 27 million more VMMC procedures by 2021, translating to 90% of males aged 10–29 years being circumcised in priority countries, will depend in part on the continued acceptability of VMMC amongst target populations.^3^ Uptake of VMMC began slowly in Botswana^9^ and has remained modest in spite of government commitment, donor support and availability of service provision of VMMC since 2009. In 2018, Botswana achieved less than 50% of the country’s target of 25 000 VMMC procedures.^10^

Studies exploring reasons for men’s unwillingness to be circumcised have identified concerns related to potential effects of VMMC on sexual function (e.g. erection and orgasm) and sexual pleasure, the risk of surgical pain, reluctance to abstain from sex for at least 6 weeks during recovery and partners’ responses.^11,12,13^ A systematic review that included > 40 000 men from cross-sectional, case-control and pre-post circumcision studies concluded that VMMC likely has little or no effect on male sexual function and satisfaction.^14^ However, ascertainment of sexual function data and findings varied across African settings^15,16,17^ and almost no data were included from the context of national VMMC programmes under ‘real-world’ conditions. One population-based cohort study in Kenya has found that the majority of the men who undergo VMMC are satisfied with the procedure and experience improvement in sexual function that increased over time.^18^ To date, no evaluations have examined sexual function amongst the men who undergo VMMC in Botswana, nor their perceptions of post-VMMC satisfaction beyond 7 days.^19^ Gathering evidence on these elements within the local context could inform VMMC messaging tailored to Botswana.

We previously assessed the frequency of adverse events at 7 days and early resumption of sexual intercourse amongst a cohort of adult men who underwent VMMC within a programmatic delivery setting in Botswana.^19,20^ This current analysis examines the post-VMMC experience at 3 months, including sexual function and satisfaction with the procedure. Given the suboptimal uptake of VMMC in Botswana and the concerns of its potentially negative impact on sexual function identified in qualitative studies,^21,22^ these new data could provide valuable evidence for VMMC demand-creation messaging in Botswana.

## Methods

### Study design and participants

We analysed data from a cohort study comprising HIV-negative, sexually active men aged 18–49 years who underwent VMMC through Botswana’s National Safe Male Circumcision programme at two government-run clinics in Gaborone, Botswana. Between November 2013 and April 2015, the parent study enrolled men to prospectively assess sexual behaviours and adverse events following VMMC.^19^ Parent study procedures have been described previously in detail.^19^ Briefly, the study was collaboratively conducted by the Botswana Ministry of Health (MOH) and the International Training and Education Center for Health (I-TECH), a collaboration between the University of Washington and the University of California, San Francisco. After individuals completed group education about the risks and benefits of VMMC and received individual counselling from clinic staff (including HIV testing), they were screened for eligibility and offered enrolment into the study. Study staff collected information on demographic, clinical, relationship and sexual behaviour characteristics at enrolment. All clinical VMMC activities were conducted per MOH guidelines at no cost to participants and were not part of the study procedures.^23^

### Data collection procedures

After circumcision, follow-up visits were scheduled in alignment with the Botswana MOH guidelines for adult VMMC (2 days, 7 days, 6 weeks, 3 months and 1 year).^23^ At study visits, participants were asked to self-administer a questionnaire about wound care, patient satisfaction of the procedure and resumption of sexual activities. At 3 months, questionnaire items included assessment of sexual functions. Each study participant was provided with a wallet-size reminder card noting the date of each follow-up visit. Prior to each visit, study staff telephoned participants to remind them of the upcoming scheduled appointment. At each follow-up visit, study staff performed a physical examination, including inspecting the circumcision site, and assessing for signs of sexually transmitted infections (STIs). Participants received BWP100 (approximately USD$8 at study initiation) at each visit as reimbursement for time and travel costs. In the event of a missed visit, study staff made telephone calls to reschedule the appointment.

### Statistical analysis

The current analysis on sexual function and satisfaction with the VMMC procedure at 3 months was restricted to men who had data available from 3-month follow-up visits, as data on earlier outcomes from this cohort had previously been reported.^23^ We identified differences between men with and without data on sexual function available at 3-month post-VMMC using Chi-square tests for proportions and Kruskal–Wallis test for continuous measures. We assessed satisfaction with the VMMC procedure and the follow-up care using a four-point Likert scale (very satisfied, somewhat satisfied, somewhat dissatisfied and very dissatisfied). We compared the frequency distributions of satisfaction at 7-day and 3-month post-VMMC amongst men who had data available from both time points to describe the changes in satisfaction with the VMMC procedure over time. We assessed whether categories of sexual function improved, had no change or worsened, compared to before undergoing VMMC using a three-point Likert scale (better, no change and worse). Domains of sexual function included sexual desire, ability to put on a condom, ease of vaginal penetration, ease of ejaculation, ability to achieve and maintain an erection, and hygiene/cleanliness. Descriptive statistics were used to summarise the frequency distributions of each sexual function category.

We evaluated the following enrolment characteristics as potential predictors of reporting any worsened sexual function: demographic information (age, education, relationship status, employment, electricity in household) and behaviour (alcohol consumption, age of sexual debut, number sexual partners [lifetime, last 12 months], type of most recent relationship [regular or casual], history of buying sex and condom use) and primary motivation for VMMC (HIV prevention vs. other reasons). Variables were identified as predictors using univariate Poisson regression models with robust error variance, an approach used when the outcome prevalence was not rare (e.g. > 10%).^24,25^ The primary outcome of our Poisson regression models was reporting any worsening of sexual function in any domain since the VMMC procedure at 3-month post-VMMC (yes or no). We included all men with 3-month follow-up data in our primary models regardless of engaging in sexual activity or not, because sexual dysfunction could influence sexual activity. In a sensitivity analysis, we restricted our models to only men who reported sexual activity after undergoing VMMC. We also reran our primary models with the outcome excluding hygiene or cleanliness. We used Stata 15/SE (Stata Corporation, College Station, TX) to perform statistical analyses.

### Ethical consideration

Ethical approvals were obtained from the Health Research and Development Committee at the Botswana Ministry of Health and Wellness (reference number: 00699) as well as the University of Washington Institutional Review Board (reference number: 42047). All participants provided written informed consent for participation in the study in addition to the consent obtained by the clinic staff for the circumcision procedure.

## Results

### Baseline characteristics

**FIGURE 1:**
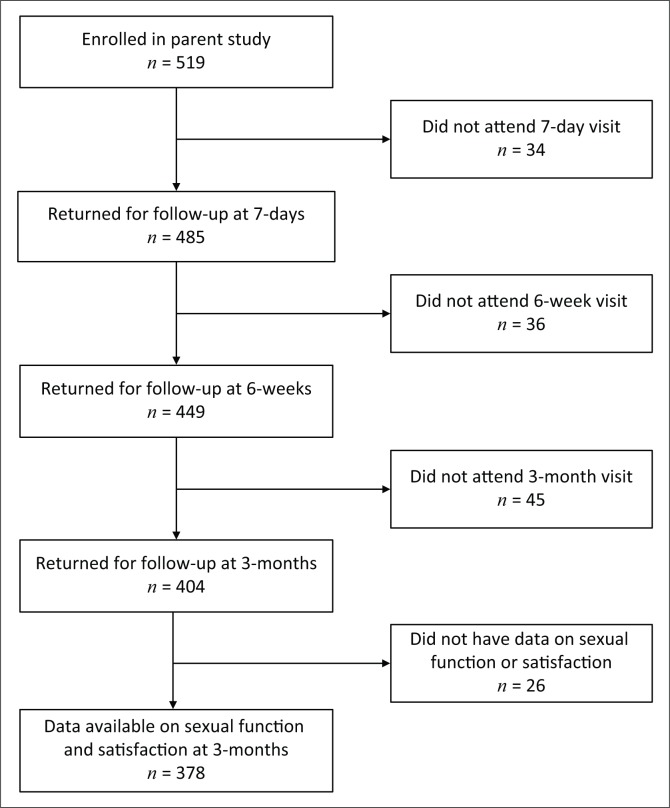
Study flowchart for men who underwent voluntary medical male circumcision and were enrolled in the parent study between November 2013 and October 2015.

In total, 519 who were enrolled in the parent study underwent VMMC (Figure 1). Median age was 27 years (interquartile range [IQR] 23–31), 57% had completed secondary education or higher and 86% had electricity in the household, a proxy for socio-economic status in this setting. The most common relationship status was dating and not living together (53%), followed by being married and/or living together (28%). Over one-third (37%) of the men reported having ≥ 6 drinks at one time at least four times a month. The median age of sexual debut was 19 years (IQR 17–20), 20% of the men reported having ≥ 2 sexual partners in the previous month and 21% reported ≥ 10 lifetime sexual partners. Almost half (47%) reported that HIV prevention was their primary reason for becoming circumcised.

Overall, 378/519 (73%) men had data available on sexual function at 3-month post-VMMC and were included in the final analysis (Figure 1). Of the 141 men without 3-month sexual function data, 82% attended a prior follow-up visit at 7 days or 6 weeks post-VMMC. Compared with men without data available, men with 3-month post-VMMC sexual function data less frequently had higher than secondary education (54% vs. 66%, *p* = 0.005). There were no differences in any other baseline characteristics between those who did have and who did not have 3-month data available (Table 1).

**TABLE 1:** Baseline characteristics of men who were uninfected by the human immunodeficiency virus who underwent voluntary medical male circumcision, by availability of sexual function data at 3-months after voluntary medical male circumcision.

Characteristic	*N* (%) of Median (IQR)						p*, ‡
	Overall *n* = 519		Sexual function data available at 3-month post-VMMC				
			Not available *n* = 141		Available *n* = 378		
	*n*	%	*n*	%	*n*	%	
**Age category (years) (*N* = 519)**							0.271
18–24	167	32.2	53	37.6	114	30.1	
25–29	175	33.7	50	35.5	125	33.1	
30–34	99	19.1	22	15.6	77	20.4	
35–40	54	10.4	12	8.5	42	11.1	
40–49	24	4.6	4	2.8	20	5.3	
**Education level (*N* = 492)**							0.005*
Primary or less	17	3.5	7	5.4	10	2.8	
Secondary	195	39.6	37	28.2	158	43.8	
Higher than secondary	280	56.9	87	66.4	193	53.4	
**Current relationship status (*N* = 509)**							0.990
Single, never married	94	18.5	25	17.7	69	18.8	
Married or living together	140	27.5	39	27.7	101	27.4	
Dating, not living together	272	53.4	76	53.9	196	53.3	
Separated, divorced or widowed	3	0.6	1	0.7	2	0.5	
**Employed (*N* = 509)**							0.450
No	150	29.5	45	31.9	105	28.5	
Yes	359	70.5	96	68.1	263	71.5	
**Electricity in household (*N* = 509)**							0.890
No	74	14.5	20	14.2	54	14.7	
Yes	435	85.5	121	85.8	314	85.3	
**Drinks alcohol (*N* = 509)**							0.750
No	197	38.7	53	37.6	144	39.1	
Yes	312	61.3	88	62.4	224	60.9	
**Has ≥ 6 drinks at least four times a month (*N* = 508)**							0.550
No	319	62.8	85	60.7	234	63.6	
Yes	189	37.2	55	39.3	134	36.4	
**Age at sexual debut, years (*N* = 502)**	19.0	17.0–20.0	19.0	17.0–20.0	19.0	17.0–21.0	0.310
**No. of sex partners (last month) (*N* = 461)**							0.170
None	153	33.2	38	30.2	115	34.3	
1 partner	217	47.1	56	44.4	161	48.1	
2+ partners	91	19.7	32	25.4	59	17.6	
**No. of lifetime sex partners (*N* = 470)**							0.950
1 partner	52	11.1	16	12.2	36	10.6	
2–4 partners	168	35.7	45	34.4	123	36.3	
5–10 partners	151	32.1	43	32.8	108	31.9	
10+ partners	99	21.1	27	20.6	72	21.2	
**Most recent sexual partner type (*N* = 459)**							0.430
Casual	49	10.7	16	12.5	33	10.0	
Regular	410	89.3	112	87.5	298	90.0	
**Always uses condom with regular partner (*N* = 299)**							0.900
No	158	52.8	47	53.4	111	52.6	
Yes	141	47.2	41	46.6	100	47.4	
**Always uses condom with casual partner (*N* = 199)**							0.870
No	57	28.6	17	27.9	40	29.0	
Yes	142	71.4	44	72.1	98	71.0	
**History of buying sex (last year) (*N* = 468)**							0.690
No	435	93.0	118	92.2	317	93.2	
Yes	33	7.0	10	7.8	23	6.8	
**Primary reason for VMMC (*N* = 509)**							0.220
Non-HIV reason	270	53.0	81	57.4	189	51.4	
HIV prevention	239	47.0	60	42.6	179	48.6	

VMMC, voluntary medical male circumcision; IQR, interquartile range; HIV, human immunodeficiency virus.

‡ , Kruskal–Wallis test for continuous measures and Chi-square test for proportions were used to detect differences in baseline characteristics between men with and without data available on sexual function at 3-month post-VMMC.

* , *p* < 0.05.

### Sexual function after voluntary medical male circumcision

**FIGURE 2:**
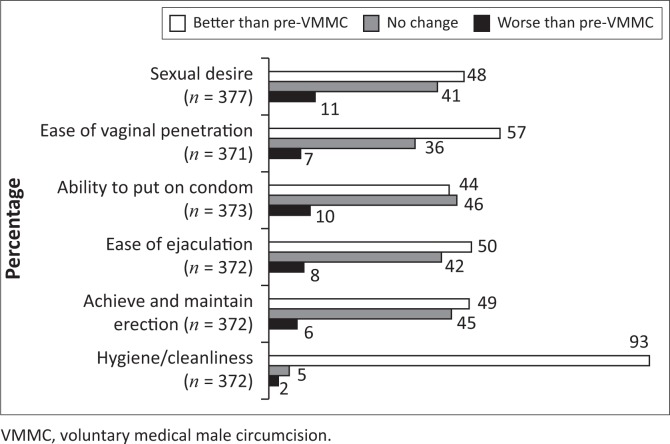
Sexual function at 3 months after voluntary medical male circumcision amongst men uninfected by the human immunodeficiency virus (*n* = 378).

Amongst men with sexual function data available at 3-month post-VMMC (*n* = 378), the majority (88%) reported having sex in the last 90 days. Amongst men who were sexually active in the last 90 days (*n* = 326), the median time since last sex was 7 days (IQR 7–14). Nearly all (96%) men reported better sexual function in at least one domain; 19% reported improvement in all six domains. The most frequently reported domain with improvement was hygiene/cleanliness (93%), followed by ease of vaginal penetration (57%); 44% – 50% of the men reported improvement in all other categories (Figure 2). One-fourth (91/378, 24%) of the men reported worsening sexual function post-VMMC in at least one domain. The most frequently reported domain to worsen was sexual desire (11%); for all other domains, less than 10% of the men reported any worsening (Figure 2). No change was reported by 36% – 44% of the men across all domains except for hygiene/cleanliness (5%). No baseline demographic, clinical or behavioural characteristics were predictive of worsening sexual function (Table 2); results were very similar in models restricted to sexually active men and when excluding hygiene/cleanliness from the domains included in the outcome (data not shown).

**TABLE 2:** Predictors of reporting worse sexual function in any category at 3 months after voluntary medical male circumcision, compared to pre-voluntary medical male circumcision amongst men uninfected by the human immunodeficiency virus who underwent voluntary medical male circumcision (*n* = 378). †, ‡

Characteristic	Worse sexual function at 3 months‡				Univariate Poisson Regression models		
	No *n* = 281		Yes *n* = 97		Crude RR	95% CI	p*
	*n*	%	*n*	%			
**Age**							
18–30 years	181	63.1	58	63.7	1.37	0.76–2.45	0.291
30–34 years	55	19.1	22	24.2	1.61	0.85–3.06	0.146
34–49 years	51	17.8	11	12.1	Ref.	-	-
**Higher than secondary education**							
No	130	46.9	38	45.2	Ref.	-	-
Yes	147	53.1	46	54.8	1.05	0.72–1.54	0.786
**Has regular partner**							
No	24	9.4	9	12.0	Ref.	-	-
Yes	232	90.6	66	88.0	0.96	0.66–1.39	0.827
**Married§**							
No	260	92.5	79	90.8	Ref.	-	-
Yes	21	7.5	8	9.2	1.18	0.64–1.20	0.595
**Lives together with partner¶**							
No	208	74.0	60	69.0	Ref.	-	-
Yes	73	26.0	27	31.0	1.21	0.81–1.79	0.349
**Employed**							
No	81	28.8	24	27.6	Ref.	-	-
Yes	200	71.2	63	72.4	1.05	0.69–1.58	0.824
**Electricity in household**							
No	42	14.9	12	13.8	Ref.	-	-
Yes	239	85.1	75	86.2	1.07	0.63–1.84	0.792
**Drinks alcohol**							
No	114	40.6	30	34.5	Ref.	-	-
Yes	167	59.4	57	65.5	1.22	0.83–1.80	0.315
**Has ≥ 6 drinks at least four times a month**							
No	175	62.3	59	67.8	Ref.	-	-
Yes	106	37.7	28	32.2	0.83	0.56–1.23	0.354
**Age at sexual debut, years**							
< 19 years	131	47.5	37	43.5	Ref.	-	-
≥ 19 years	145	52.5	48	56.5	1.13	0.78–1.64	0.526
**No. of sex partners (last month)**							
< 2 partners	217	84.1	59	76.6	Ref.	-	-
≥ 2 partners	41	15.9	18	23.4	1.43	0.91–2.23	0.119
**No. of lifetime sex partners**							
< 5 partners	120	46.9	39	47.0	Ref.	-	-
≥ 5 partners	136	53.1	44	53.0	1.00	0.68–1.45	0.986
**Most recent sexual partner type**							
Casual	24	9.4	9	12.0	Ref.	-	-
Regular	232	90.6	66	88.0	0.81	0.45–1.48	0.495
**Inconsistent or no condom use with regular partner**							
No	84	51.9	27	55.1	Ref.	-	-
Yes	78	48.1	22	44.9	0.90	0.55–1.48	0.691
**Inconsistent or no condom use with casual partner**							
No	29	30	11	26	Ref.	-	-
Yes	67	70	31	74	1.15	0.64–2.06	0.638
**History of buying sex (last year)**							
No	246	93.5	71	92.2	Ref.	-	-
Yes	17	6.5	6	7.8	1.16	0.57–2.39	0.678
**Primary reason for VMMC**							
Non-HIV reason	141	50.2	48	55.2	Ref.	-	-
HIV prevention	140	49.8	39	44.8	0.86	0.59–1.24	0.417

RR, risk ratio; VMMC, voluntary medical male circumcision; CI, confidence interval.

† , Includes men who had data available on sexual function at 3-month post-VMMC procedure.

‡ , Having worse sexual function at 3-month post-VMMC, compared to pre-VMMC in any of the following categories: sexual desire, ease of vaginal penetration, ability to put on a condom, ease of ejaculation, achieve and maintain erection, hygiene/cleanliness.

§ , Includes married men who live or do not live with their wives.

¶ , Includes married men who live with their wives or unmarried men who live with their partners.

* , *p* < 0.05; Wald test *p*-value for categorical variables.

### Satisfaction with voluntary medical male circumcision procedure

**FIGURE 3:**
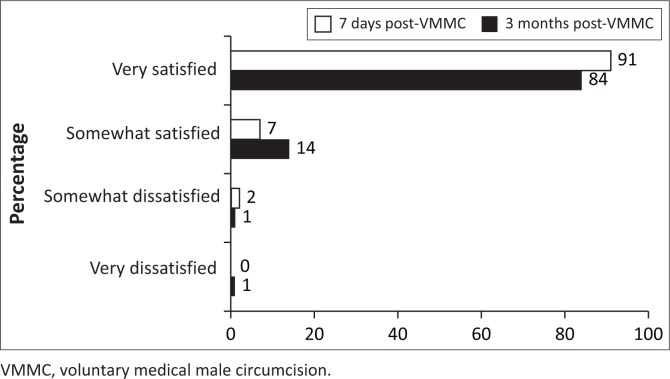
Satisfaction with voluntary medical male circumcision procedure at 7 days and 3 months after voluntary medical male circumcision amongst men who had data available at both time points (*n* = 375).

**FIGURE 4:**
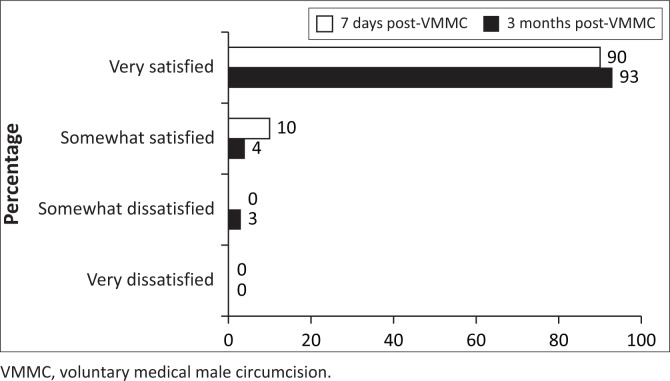
Satisfaction with voluntary medical male circumcision follow-up care at 7 days and 3 months after voluntary medical male circumcision amongst men who had data available at both time points (*n* = 375).

Overall, 84% of men were very satisfied with the VMMC procedure at 3 months, 14% were somewhat satisfied, 1% were somewhat dissatisfied and 1% were very dissatisfied. Frequency of being very satisfied with the VMMC procedure was slightly lower at 3 months, compared with 7-day post-VMMC procedure (Figure 3, 84% vs. 90%, *p* = 0.004). Almost all (93%) of the men reported being very satisfied with follow-up care at 3 months (Figure 4). Amongst men who were very satisfied with VMMC at 7 days and became less satisfied/dissatisfied at 3 months (*n* = 23), 17% reported worsening sexual function. At 3 months, men who reported any worsening of sexual function were 2.3-fold as likely to be less than ‘very satisfied’ with the VMMC procedure at 3 months (risk ratio 2.36, 95% confidence interval [CI] 1.66–3.34, *p* < 0.001). Amongst men who were overall somewhat or very dissatisfied with VMMC at 3 months (*n* = 7), non-mutually exclusive reasons for being dissatisfied included appearance (*n* = 4), wound care requirements (*n* = 2), aspects of the procedure (*n* = 3) and issues with pain (*n* = 2).

## Discussion

In this longitudinal evaluation of the men who became circumcised within a programmatic VMMC setting in Botswana, we found very high overall satisfaction with the procedure at 3 months after VMMC accompanied by frequently reported improvement in sexual function. Consistent with prior data from research settings, our implementation evaluation found that nearly all (98%) men were at least somewhat satisfied overall with the VMMC procedure; almost one-fifth (19%) reported improved sexual function in every category assessed; and over one-third reported no change.^26^ Although few men reported being dissatisfied with VMMC, frequency of worsening sexual function post-VMMC was higher in this group. Our results highlight considerations for demand-creation messaging as VMMC programmes continue to roll out in countries such as Botswana that have high HIV burden and modest VMMC uptake.

Similar to prior studies that evaluated sexual function pre- and post-circumcision amongst African men,^15,18^ we found that the majority of the men reported improvement in some domains of sexual function after undergoing VMMC in Botswana. When asked to retrospectively compare with the condition before undergoing VMMC, approximately half of the men in our evaluation reported improved sexual desire, ease of vaginal penetration and ejaculation, and/or ability to achieve and maintain an erection post-VMMC. Studies amongst men who became circumcised in adulthood are especially useful in evaluating the impact of VMMC on sexual function because these men served as their own control.^14^ Data from randomised trials in Uganda and Kenya^15,16^ provide the highest quality evidence on the effects of VMMC on sexual function and found some improvement in sexual function after VMMC. There are important contextual differences between men who were willing to enrol in the early VMMC trials, when efficacy was unknown and VMMC was randomly assigned, and those who self-select VMMC delivered as part of national programmes. Our findings contribute to the evidence base supporting the benefits of VMMC in programmatic settings extended beyond HIV prevention and could broadly improve sexual health amongst African men.^17^ The future programmatic evaluations could improve design rigour by assessing sexual function prior to undergoing VMMC and comparing it with post-VMMC assessment.

In spite of the high levels of improvement in sexual function following VMMC, an appreciable proportion (19%) of the men in our evaluation reported at least one category of sexual function worsening after VMMC. A systematic review published in 2013 by Morris and Krieger^14^ that included 20 931 circumcised men and 19 542 uncircumcised men found no evidence for differences in any component of sexual function by circumcision status. However, almost no data were included from programmatic delivery settings in African countries that could provide different quality of services or reach a population unlike men enrolled in the initial VMMC randomised trials.^15,16^ In addition, prior randomised trials^15,16^ assessed prevalence of sexual dysfunctional components (erectile dysfunction, ease of ejaculation, etc.) up to 24 months post-VMMC and found a decrease in dysfunction over time. In our programmatic evaluation, we asked participants to retrospectively report changes in sexual functioning, if any, at the 3-month follow-up visit (as opposed to conducting a separate assessment of sexual functioning at baseline). Men who were less satisfied with the VMMC procedure were also more likely to report worsening of sexual function. Men currently experiencing sexual dysfunction may be more likely to report that their status had worsened after VMMC because of recall bias. It is also possible that men enrolled in our evaluation could experience better sexual function at later time points. We also found modestly lower, although still very high, satisfaction with the VMMC procedure at 3 months (84%), compared to 7-day (90%) post-VMMC. To our knowledge, no other programmatic evaluations amongst African men have evaluated satisfaction with VMMC over time. The future programmatic evaluations could identify areas for improving the VMMC procedure, counselling and follow-up care to prevent potential negative effects on sexual function and satisfaction with the procedure.

Over a decade after initiation, uptake of VMMC in Botswana’s national programme remains modest.^10^ A recent systematic review from July 2019 by Ensor et al. has found that the most effective VMMC demand-creation interventions are financial incentives and education or counselling programmes delivered by community opinion leaders or individuals with personal experience of VMMC.^27^ To date, no VMMC demand-creation intervention studies have been conducted in Botswana and ongoing mass media campaigns focus on protection against HIV associated with VMMC. The future demand-creation messages in Botswana could be tailored to the values and preferences of the men at risk of HIV infection whilst extending beyond HIV prevention.^28,29^ Integrating evidence for improved sexual function and satisfaction following VMMC into mainstream messaging could potentially motivate Batswana men, who may not perceive their own HIV risk or for whom HIV prevention is not a motivator for VMMC, to seek VMMC. Our evaluation found high frequency of improved sexual function and overall satisfaction with VMMC within programmatic settings, which could be helpful for framing the holistic benefits of VMMC in future messaging.

Our study has limitations. We only ascertained sexual function information at 3-month post-VMMC and retrospectively asked men to compare aspects of sexual function with their pre-VMMC experiences. This approach was intended to capture how VMMC impacts sexual function, although a baseline assessment of sexual function would have improved the rigour of our findings. Recall bias is possible with over-reporting of worsening sexual function amongst dissatisfied men or potentially choice-supportive bias of improved function to rationalise the decision to undergo VMMC. Not all men returned for follow-up and/or had data on sexual function available at 3-month post-VMMC. This may bias our overall estimates of satisfaction and sexual function. We detected minimal baseline differences between men with and without 3-month data available, and no characteristics were predictive of worsening sexual function. Men who did not return for follow-up visits could potentially be more or less satisfied with their VMMC experience, which could bias our results. We intentionally conducted an implementation evaluation without intensive follow-up tracing to align with programmatic settings. The future evaluations could include more research procedures such as at-home follow-up tracing to collect data that are more complete.

## Conclusion

To date, demand-creation messaging for VMMC in Botswana has primarily focussed on HIV prevention. However, as VMMC uptake remains modest amongst Batswana men, there is a need to effectively promote VMMC to men at-risk of HIV infection for whom protection against HIV may not be a motivator to undergo VMMC. Similar to prior studies from research settings in other African countries, our programmatic data show that the majority of the men report improved sexual function across multiple categories and high overall satisfaction after VMMC. As VMMC continues to roll out in Botswana, incorporating evidence of other non-HIV-related benefits of VMMC into demand-creation messaging may support maximising VMMC uptake.
